# Kinematics and center of axial rotation during walking after medial pivot type total knee arthroplasty

**DOI:** 10.1186/s40634-020-00286-y

**Published:** 2020-09-28

**Authors:** Kota Miura, Yasumitsu Ohkoshi, Takumi Ino, Kengo Ukishiro, Kensaku Kawakami, Sho’ji Suzuki, Ko Suzuki, Tatsunori Maeda

**Affiliations:** 1 Department of Rehabilitation, Hakodate Orthopedics Clinic, 2-115, Ishikawa-cho, Hakodate-shi, Hokkaido 041-0802 Japan; 2 Department of Orthopedic Surgery, Hakodate Orthopedics Clinic, Hakodate, Japan; 3 Department of Physical Therapy, Faculty of Health Sciences, Hokkaido University of Science, Sapporo, Japan; 4 Department of Production Systems Eng., National Institute of Technology, Hakodate College, Hakodate, Japan; 5 Department of Complex and Intelligent Systems, Future University Hakodate, Hakodate, Japan

**Keywords:** Lateral pivot, Total knee arthroplasty, Center of axial rotation, Kinematics, Walking

## Abstract

**Purpose:**

In recent years, the medial pivot (MP) type total knee arthroplasty (TKA) implant has been developed and marketed for achieving more natural kinematics with MP. However, little is known about the pivot pattern during walking after MP type TKA. This study aimed to determine the kinematics and center of axial rotation during walking after MP type TKA.

**Methods:**

This randomized prospective study enrolled 40 patients with MP type TKA, 20 with cruciate-substituting TKA (MP-CS group), 20 with posterior-stabilized TKA (MP-PS group), and 10 healthy volunteers (control group). The kinematics and center of axial rotation during overground walking were measured by a three-dimensional motion analysis system. The six-degrees-of-freedom kinematics of the knee were calculated by the point cluster method.

**Results:**

The amount of change in knee flexion in early stance phase was significantly lower in the MP-CS and MP-PS groups than in the control group. The femur showed anterior translation during early stance phase in all three groups. The median center of axial rotation in the transverse plane was predominantly on the lateral side of the knee during stance in all groups.

**Conclusions:**

Kinematics during gait are thought to be determined by physical posture, the kinetic chain during weight-bearing, and the kinematic features of adjacent structures, such as the behavior of the biarticular muscles. MP-CS and MP-PS did not necessarily induce rotational motion centered on the medial ball-in-socket component during walking; translational and lateral pivoting movements were also observed. Long-term follow-up is needed to monitor for polyethylene wear and implant loosening.

## Background

Six-degrees-of-freedom (6DOF) kinematics of the knee joint during walking are determined by physical posture, the position of the body’s barycenter, the kinetic chain during weight-bearing, muscle coordination and antagonism, and the kinematic features of adjacent structures, including the behavior of the biarticular muscles [2, 8, 12, 15]. In recent years, many types of implants for total knee arthroplasty (TKA) have become available. The center of axial rotation (COR) of the knee joint is expressed as either medial or lateral pivot depending on the position of the COR on the tibial plateau [3]. Medial pivot (MP) type TKA is designed to create a medial ball-in-socket structure to ensure a medial location of the COR for medial pivot motion. Several motion analysis studies [4, 21–23] have been reported for MP type TKA; however, the 6DOF kinematic features of the knee joint when overground walking after surgery have yet to be fully elucidated. Moreover, the question of whether medial pivot motion is physiological remains controversial. Several fluoroscopic studies [6, 7, 10, 13, 15] of walking and lunge motions in both living human knees and cadaveric knees show medial pivot motion at the knee. By contrast, a fluoroscopic study by Kozanek et al. [17] found that lateral pivot occurred during walking in healthy subjects. Furthermore, Koo et al. [16] analyzed walking in healthy subjects by using an optical motion capture technique and reported the presence of lateral pivot motion. The findings of these reports are inconsistent with the position of the COR, even in healthy knees. The present study aimed to determine the 6DOF knee kinematics and location of the COR in the transverse plane of the knee during walking in patients with MP type TKA.

## Methods

**Fig. 1:**
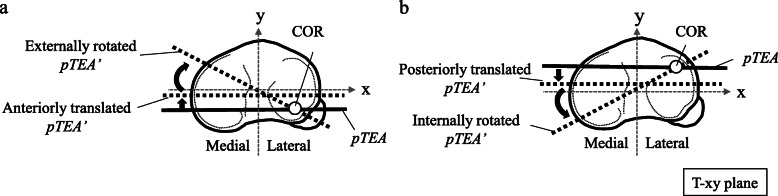
COR estimation during walking. The projected transepicondylar axis of the femur on the tibial x-y plane was defined as the pTEA (solid line). The pTEA’ (dotted line) was defined as the change in pTEA after knee motion. The COR was defined as the intersection of pTEA and pTEA’. When the pTEA translated anteriorly and rotated externally, the COR was lateral, that is, lateral pivot (**a**). When the pTEA translated posteriorly and rotated internally, the COR was lateral (**b**). COR, center of axial rotation

Patients who underwent TKA using the Evolution® Medial Pivot Knee System (MicroPort Orthopedics Inc., Arlington, VA) at our hospital from February 2015 to February 2016 were randomly allocated (1:1) using sequentially numbered, opaque, sealed envelopes to a cruciate-substituting (MP-CS) group or a posterior-stabilized (MP-PS) group in a prospective manner. The inclusion criteria were 1) age 20 years or older; 2) osteoarthritis (OA) of the knee with a Kellgren-Lawrence grade of ≥2; and 3) a femorotibial angle (FTA) of < 190° on a radiographic frontal view in the standing posture (range of FTA, 172.0°–189.9°). Patients with rheumatoid arthritis, lateral type OA with valgus alignment, or a limp were excluded. Applying these criteria, 20 knees of 20 patients undergoing MP-CS TKA (1 male, 19 female) and 20 knees of 20 patients undergoing MP-PS TKA (2 male, 18 female) were included in the study. Two patients in whom a postoperative evaluation was not possible (1 with lumbar disease and 1 with traumatic patellar tendon rupture following MP-CS TKA) were subsequently excluded. Two further patients (1 in the MP-CS group and 1 in the MP-PS group) could not attend the postoperative evaluation for social reasons. Clinical evaluations were performed before and 1 year after surgery. The motion analyses were performed 1 year after surgery in 17 knees of 17 patients (1 male, 16 female, mean age 71.6 ± 5.5 years, body mass index [BMI] 27.4 ± 4.9) in the MP-CS group and in 19 knees of 19 patients (2 male, 17 female, mean age 73.1 ± 5.5, BMI 26.8 ± 3.0) in the MP-PS group. The control group comprised 10 knees of 10 healthy volunteers (4 male, 6 female, mean age 27.8 ± 4.7 years, BMI 20.0 ± 2.0). The clinical items evaluated were range of motion (ROM) and plain standing radiographs. The 2011 Knee Society Score (KSS 2011) was recorded. The α, β, γ, and δ angles, the FTA, and the WBL ratio were also measured. The medial and lateral joint space widths were measured on varus and valgus stress radiographs using a Telos SE stress device (Telos GmbH, Hungen, Germany). After preoperative planning, surgery was performed by two experienced orthopedists using the same measured resection technique. In all cases, patella replacement and posterior cruciate ligament resection were performed. No medial release was performed and only medial bony spurs were resected. Three-dimensional (3D) motion analysis of walking was performed in all three groups. The 3D motion analysis system consisted of 8 infrared cameras (ProReflex, Qualisys AB Inc., Gothenburg, Sweden) and 2 force plates (OR6; Advanced % Technology Inc., Watertown, MA). The measurement frequency of the infrared cameras and force plates was set at 120 Hz. To avoid mutual interference, the force plates were arranged independently of each other in the halfway zone of the 8-m walking pathway such that subjects could not recognize the sites of the plates. Each subject walked barefoot on the pathway with a steady gait at a self-selected comfortable walking speed. Measurement was considered complete upon achieving three successful walking cycles. To avoid the influence of fatigue, each subject rested adequately between cycles. Skin markers were attached to the lower limbs (56 markers) and to the acromion on both sides by a physical therapist with detailed knowledge of the surface anatomy of the knee using the point cluster (PC) method [2, 12]. The Qualisys Track Manager 3D motion analysis software program was used to analyze the recorded data by the PC method. One walking cycle was defined as the interval between heel strike of a foot to the next heel strike of the same foot, standardized as 100%, based on information from the infrared cameras synchronized with the floor reaction force sensors. The joint coordinate system for the PC method was set based on the definition of Grood and Suntay [11]. In the tibial coordinate system, the mediolateral axis of the tibia (x axis) was defined as a line segment connecting the points several millimeters proximal to the apices of the medial and lateral condyles of the tibia (i.e., points consistent with the articular surface). The line segment on the articular surface passing through the midpoint of the aforementioned line segment and perpendicular to the x axis was defined as the anteroposterior axis (y axis). The intersection of the x and y axes was regarded as the origin, and a line passing through the origin and orthogonal to the x-y plane was regarded as the z axis. In the femoral coordinate system, the line segment connecting the medial and lateral epicondyles of the femur was defined as the transepicondylar axis (TEA), and the midpoint of the TEA was regarded as the origin. The line passing through the origin and parallel to the long axis of the femur was defined as the supra-inferior axis, and the line passing through the origin and perpendicular to the TEA and the supra-inferior axis was defined as the anteroposterior axis. The kinematics of the knee during walking, shown as movement of the femur with respect to the tibia, were then analyzed. The flexion and extension angles of the knee joint and the rotation and anteroposterior translation movement of the femur were examined. The knee kinematics were analyzed by setting the angle and location of the standing position at rest with the knee fully extended as point zero. The projected transepicondylar axis (pTEA) of the femur in the tibial x-y plane was defined as the pTEA. The change in pTEA after knee motion was defined as the pTEA’. The COR was defined as the intersection of pTEA and pTEA’ (Fig. 1). The instantaneous COR (ICOR) was calculated per 0.0083 s during walking, and all x coordinates of the ICOR from three trials were assessed in histograms.

The unpaired *t*-test was used to compare the clinical findings between the MP-CS and MP-PS groups. The Mann-Whitney *U* test was used to compare the postoperative KSS 2011 results between the two groups. The significance level was set at less than 5%. The Kruskal-Wallis test was used to compare peak values and amounts of change in the knee kinematics and the location of median COR among the three groups with the Dunn-Bonferroni method used for post hoc testing, with the significance level set at *P* < 0.017.

The ethics committee of our institution approved this study, and all subjects provided written informed consent after receiving a detailed explanation of the study.

## Results

There was no significant difference in preoperative knee flexion angle, bone morphology angle, or clinical outcome between the MP-CS and MP-PS groups (Table 1). The walking speed was 1.4 m/s in the control group. Walking speeds were significantly lower in the MP-CS and MP-PS groups both pre- and postoperatively compared with the control group (Table 2). There was no significant difference in postoperative ROM between the MP-CS group and the MP-PS group (130.3 ± 7.0° vs 133.4 ± 6.7°). Furthermore, there were no significant differences in radiographic alignment or findings on stress radiography between these two groups (Table 3). The KSS 2011 scores were significantly better in the MP-CS group than in the MP-PS group in terms of patient satisfaction with current knee pain of major category II both overall and for the following specific items: 1, sitting in a chair; 3, getting out of bed; 4, light housework; and 5, going out or other recreational activity. There were no significant differences in any of the other items (Table 4).

**Table 1:** Comparison of preoperative knee flexion angle, bone morphologic angles, and clinical outcome

Group	Knee flexion (°)	FTA (°)	α angle (°)	β angle (°)	γ angle (°)	δ angle (°)	WBL (%)	Medial dilation of the tibiofemoral joint (mm)	KSS 2011 (total)
MP-CS	132.5 ± 9.8	183.0 ± 4.6	98.5 ± 2.4	84.0 ± 3.3	–	81.8 ± 2.9	9.0 ± 19.3	6.7 ± 1.0	84.9 ± 23.2
MP-PS	137.5 ± 10.7	179.8 ± 4.4	98.9 ± 1.6	84.5 ± 2.2	–	80.5 ± 3.1	21.4 ± 16.3	7.2 ± 1.0	82.9 ± 23.0

*FTA*, femorotibial angle; α, angle of the distal femoral end; β, angle of the proximal tibial end; γ, femoral flexion angle; δ, posterior tibial slope; *WBL* Weight bearing line; *KSS 2011* 2011 Knee Society Score; *MP* Medial pivot; *CS* Cruciate-substituting; *PS* Posterior-stabilized. There was no significant difference between the two MP groups (unpaired *t*-test and Mann-Whitney *U* test, significance level < 5%)

**Table 2:** Comparison of preoperative and postoperative walking speed

		Before surgery		After surgery
Walking speed(m/s)	MP-CS group	0.9 ± 0.2*^†^		1.0 ± 0.1*^§^
	MP-PS group	1.1 ± 0.2*^†^		1.0 ± 0.2*^§^
	Control group		1.4 ± 0.1	

*MP* Medial pivot; *CS* Cruciate substituting; *PS* Posterior-stabilized

*P* < 0.05, Kruskal-Wallis test,

*Significantly slower than in the control group

^†, §^not statistically significant

**Table 3:** Comparison of knee flexion angle and radiographic measurements at 1 year after surgery

	Knee flexion (°)	FTA (°)	α angle (°)	β angle (°)	γ angle (°)	δ angle (°)	WBL (%)	Medial dilation of the tibiofemoral joint (mm)
MP-CS group	130.3 ± 7.0	176.3 ± 2.7	96.5 ± 1.9	88.5 ± 1.5	2.1 ± 1.9	84.7 ± 2.4	40.0 ± 7.2	1.4 ± 0.7
MP-PS group	133.4 ± 6.7	175.2 ± 2.5	97.0 ± 1.7	88.7 ± 1.5	3.0 ± 2.6	84.0 ± 2.1	40.8 ± 13.3	1.6 ± 0.5

*FTA* Femorotibial angle; α, femoral component angle; β, tibial component angle; γ, femoral component flexion angle; δ, tibial posterior slope; *WBL* Weight bearing line; *MP* Medial pivot; *CS* Cruciate-substituting; *PS* Posterior-stabilized. There was no significant difference between the two MP groups (unpaired *t*-test, significance level < 5%)

**Table 4:** Comparison of satisfaction with each item and the total KSS 2011 at 1 year after surgery

Type	1 Sitting in a chair	2 Lying in bed	3 Getting out of bed	4 Light housework	5 Going out, recreation	II total	KSS 2011
CS group	6.1 ± 1.5*	5.5 ± 1.8	5.6 ± 1.8*	5.5 ± 1.5*	5.5 ± 1.7*	28.4 ± 7.5*	130.8 ± 20.9
PS group	4.9 ± 1.5*	4.8 ± 1.7	4.3 ± 1.7*	4.2 ± 1.6*	3.7 ± 1.7*	22.0 ± 7.1*	122.3 ± 25.5

*p* < 0.05, Mann-Whitney *U* test

*Significant difference. KSS 2011, Knee Society Score (2011); *CS* Cruciate-substituting; *PS* Posterior-stabilized

**Fig. 2:**
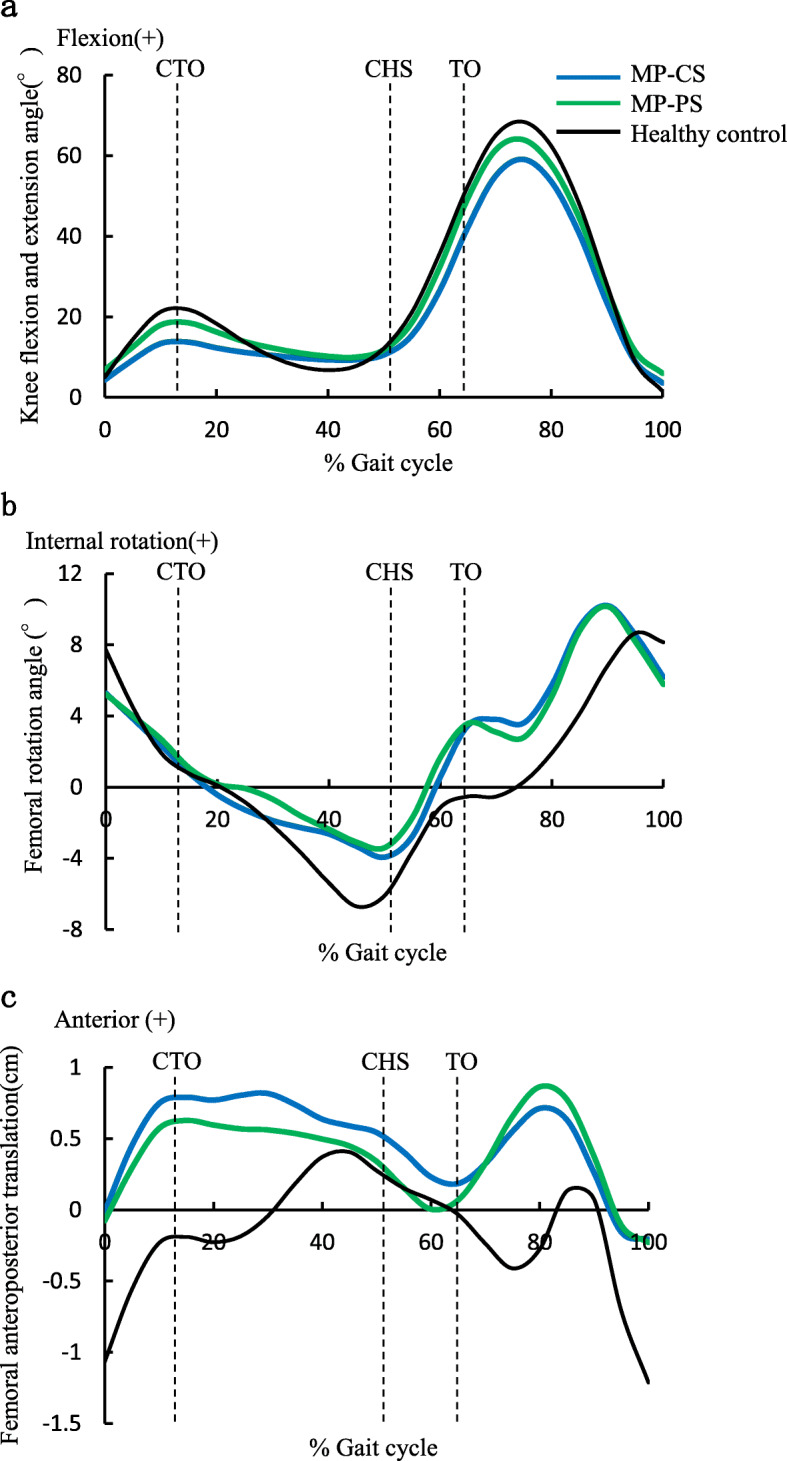
Kinematics of the knee joint during walking. All three groups showed knee flexion twice during one walking cycle and external rotation in stance phase (**a**, **b**). In both the MP-CS and the MP-PS groups, the femur showed anterior translation during early stance phase, maintained the anterior position during mid-stance phase, and showed posterior translation during late stance phase to pre-swing phase (**c**). CTO, contralateral toe-off; CHS, contralateral heel strike; TO, toe-off; MP, medial pivot; CS, cruciate-substituting; PS, posterior-stabilized

All three groups showed knee flexion twice during one walking cycle and the femur rotated externally from early to mid-stance and then rotated internally (Fig. 2a, b). In the control group, the femur showed anterior translation during early stance phase, followed by maintenance of the anterior position or slight posterior translation, then another anterior translation, and finally posterior translation during late stance phase to swing phase. However, in both the MP-CS and the MP-PS groups, the femur showed anterior translation during early stance phase, maintained the anterior position during mid-stance phase, and showed posterior translation during late stance phase to swing phase (Fig. 2c).

Quantitative analysis of the knee kinematics during walking revealed that the amount of change in knee flexion in early stance phase was significantly lower in the MP-CS and MP-PS groups than in the control group (9.6° and 12.1°, respectively, vs 17.0°). The femur in stance phase was externally rotated in all three groups, reaching its peak almost simultaneously with peak extension of the knee. The mean angle of maximum external rotation was 4.7° in the MP-CS group, 4.1° in the MP-PS group, and 6.9° in the control group, with a significant difference between the MP-PS group and the control group. The amount of anterior translation of the femur in stance phase was 8.0 mm in the MP-CS group, 7.3 mm in the MP-PS group, and 8.8 mm in the control group, with no significant difference among the groups.

**Fig. 3:**
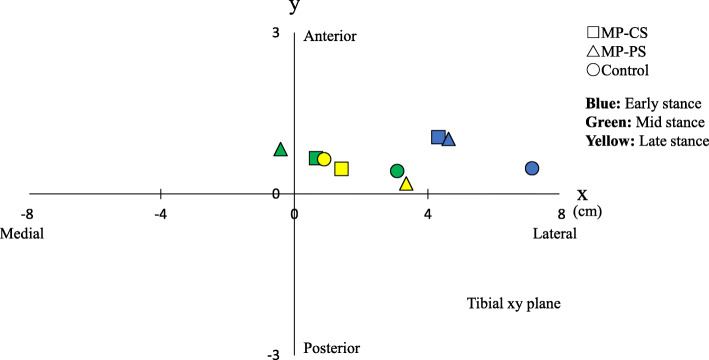
Median COR during early stance, mid-stance, and late stance to swing phase of gait. Square, MP-CS group; triangle, MP-PS group; circle, control group. Blue indicates early stance, green indicates mid-stance, and yellow indicates late stance to pre-swing of gait. COR, center of axial rotation; MP, medial pivot; CS, cruciate-substituting; PS, posterior-stabilized

**Fig. 4:**
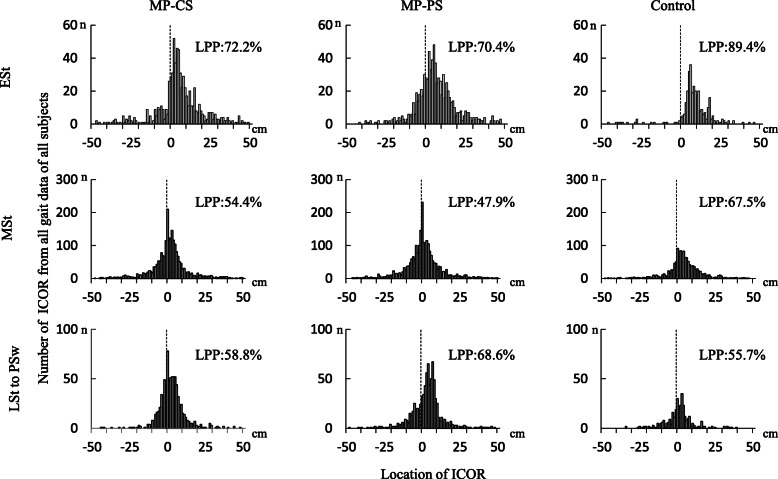
Distribution of all x-coordinates of the ICOR in each phase of gait. The “0” on the horizontal axis indicates the center of the tibial plateau. The LPP indicates lateral pivot proportion (%). ICOR, instantaneous center of axial rotation; ESt, early stance; MSt, mid-stance; LSt, late stance; PSw, pre-swing

**Fig. 5:**
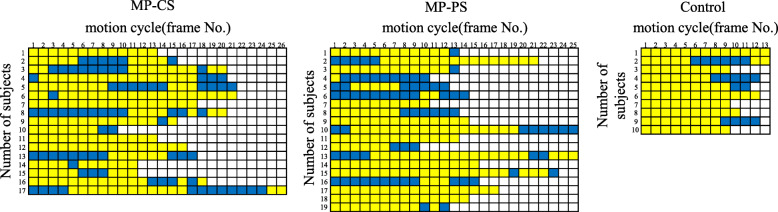
Changes over time in location of the ICOR in early stance of gait. Blue indicates medial pivot and yellow indicates lateral pivot. The first frame number indicates heel strike and the last frame number indicates contralateral toe-off in each subject. ICOR, instantaneous center of axial rotation

The median CORs (x [cm], y [cm]) in early stance were (4.3, 1.1) in the MP-CS group, (4.6, 1.0) in the MP-PS group, and (7.1, 0.5) in the control group. In all groups, the COR was located in the first quadrant (lateral side). The x-coordinate was significantly smaller and more medial in both TKA groups than in the control group. During mid-stance, the median CORs were (0.7, 0.7) in the MP-CS group, (− 0.4, 0.8) in the MP-PS group, and (3.1, 0.4) in the control group; the COR in the MP-PS group was located in the medial quadrant. During late stance, the median CORs were (1.4, 0.5) in the MP-CS group, (3.4, 0.2) in the MP-PS group, and (0.9, 0.6) in the control group; the x-coordinates were located on the lateral side in all groups (Fig. 3). Histogram analysis of the ICORs revealed that they were lateral in 72.2% of subjects in the MP-CS group, in 70.4% in the MP-PS group, and in 89.4% in the control group during early stance, 54.4% in the MP-CS group, 47.9% in the MP-PS group, and 67.5% in the control group during mid-stance, and in 58.8% in the MP-CS group, 68.6% in the MP-PS group, and 55.7% in the control group during late stance to pre swing (Fig. 4). Over time, some knees showed lateral pivot only or coexistence of medial and lateral pivot during early stance in all three groups (Fig. 5).

## Discussion

The control group in this study showed external rotation and anterior translation of the femur in stance phase. Lafortune et al. [18] analyzed knee kinematics in healthy subjects by an optical motion capture technique using metal pins with an optical reflection marker inserted into the tibia and femur. They reported that the femur showed external rotation and anterior translation twice during stance. Koo et al. [16] analyzed the kinematics and ICOR of 23 healthy knees using the PC method and reported that all the average ICORs were located laterally due to the femur underwent external rotation and anterior translation in stance phase during walking. Kozanek et al. [17] analyzed movement of the femur in healthy subjects using biplane radiography and magnetic resonance imaging and reported external rotation and anterior translation in early stance phase during walking. The results of these three studies are consistent with those of our study, indicating the validity of our analyses of knee kinematics.

Quantitative analyses of knee flexion and extension during walking showed that the amounts of change in early stance phase and mid-stance phase were significantly lower in the MP-CS and MP-PS groups than in the control group. McClelland et al. [20] compared knee kinematics during comfortable walking between patients 1 year after TKA and 40 healthy subjects matched for age and sex and reported that the knee flexion angles in both stance and swing phases were significantly smaller in the patients who had undergone TKA, suggesting that control by the quadriceps and hip extensor muscles in loading response remained inadequate 1 year after surgery. A similar phenomenon may have occurred in the patients in the MP-CS and MP-PS groups in this study, whose postoperative walking speed was significantly lower than that of subjects in the control group. This slow walking pace may also have contributed to the small angle of knee flexion.

External rotation and anterior translation of the femur were observed in stance phase during walking in the MP-CS and MP-PS groups. However, in both these groups, anterior translation occurred in early stance phase. It is thought that the breaking action of the tibia causes the femur to slide forward as the knee flexes [17]. Anterior translation of the femur was maintained in mid-stance phase in the MP-CS and MP-PS groups, and the maximum external rotation angle of the femur in stance phase was significantly smaller in the MP-PS groups than that in the control group. This phenomenon resembles the compensatory movement commonly observed in walking by patients with anterior cruciate ligament (ACL) insufficiency [5, 8], which presumably suppresses the anterolateral rotatory instability and anterior displacement of the tibia. Given that knees treated by TKA are also ACL-insufficient, it is possible that similar compensatory movements occurred in the patients in this study. This possibility requires further investigation involving moment analysis.

Whether or not MP always induces medial pivot motion in the ball-in-socket structure of the medial tibiofemoral joint remains an important issue. As mentioned previously, there was anterior translation and simultaneous external rotation of the femur in early stance phase during walking in both the MP-CS and MP-PS groups. Analysis of this motion in terms of COR revealed lateral pivot (Fig. 1-a). The femur showed posterior translation, which indicates roll back, from late stance to swing phase, and internal rotation of the femur was simultaneously present. Simultaneous occurrence of posterior translation and internal rotation of the femur means lateral pivot when analyzed in terms of COR (Fig. 1-b). Furthermore, all three groups included subjects showing lateral pivot only or coexistence of medial and lateral pivot changing over time in location of the ICOR during early stance (Fig. 5). These results suggest that, in both the MP-CS and MP-PS groups, medial pivot was not constantly induced in stance phase during walking, with lateral pivot being present instead. Banks and Hodge reported that PS and cruciate-retaining (CR) implants showed lateral centers of rotation in stance phase during walking [3]. These provides a view of the COR is not located at a single point because COR is mainly determined by knee kinematics, especially translation and rotation, which depends on the type of motion, implant design, and characteristics of individuals. Therefore, COR kinematics vary according to the type and phase of motion and among individuals, and the COR seemed to vary according to these kinematic differences. The ball-in-socket structure of the medial joint in the MP-CS knee does not necessarily induce medial pivot motion, and it is thought that translational and lateral pivot motion may occur. In particular, the occurrence of translation and lateral pivot motion in MP-CS indicates the possibility of overriding of the femoral implant on the tibial socket margin. This shows that the ball-in-socket structure of the MP-CS functions as a restraint mechanism in the anteroposterior direction; however, if overriding of the femoral implant occurs, then there is a concern about wear and breakage of the polyethylene material in the long term. A good outcome for MP 10 years after surgery has been reported previously [14, 19]. However, this result may be due to improvements in the quality of polyethylene inserts. Moreover, better conformity of the inserts has resulted in more anterior-posterior translation, higher wear rates, and a greater wear area [9, 24]. Therefore, further long-term follow-up is required.

Postoperative satisfaction rated using the KSS (2011) was significantly better in the MP-CS group than in the MP-PS group. This was thought to be because the surface shape of the MP-CS was preferred by patients when performing activities of daily living that induce medial pivot, such as standing up from a seated position, sitting down in a chair, and deep knee bending [15].

This study has several limitations. First, the participants in both MP groups were older than those in the control group and their walking speed was slower. The possibility that these limitations affected kinematics during walking cannot be excluded. Furthermore, patients with severe varus and valgus knee OA were excluded because the kinematics of knees with severe varus or severe valgus OA is significantly different from those of knees with general OA. Accordingly, if severe OA-related deformity were concentrated in one group, it would likely be a confounding factor. Moreover, measurement error is a common problem with optical motion capture using skin markers. Alexander et al. [1] calculated the measurement error of the PC method using an external fixator device and reported that the maximum error was 4° for rotation and 3 mm for translation, indicating the need for caution when interpreting changes smaller than these values. However, as mentioned previously, the findings for the kinematics of walking in healthy controls in our present study were qualitatively consistent with those of previous studies that used different methodologies [16–18]. This indicates that the PC method is suitable for analyzing walking.

In summary, this study found a small change in flexion and extension angles, anterior translation of the femur in early to mid-stance phases, and slight or no internal rotation of the tibia in the slight knee flexion position in stance phase during gait in knees that had undergone MP-CS or MP-PS. These findings resemble the compensatory motion observed in knees with ACL insufficiency. Moreover, MP-CS and MP-PS did not necessarily induce rotational motion centered on the medial ball-in-socket component during walking; rather, translational and lateral pivoting motions were observed. Therefore, the influence of MP surface geometry on kinematics during walking is thought to be limited.

## Conclusion

In this study, the majority of patients who underwent medial pivot TKA showed the lateral pivot pattern during walking. For MP-CS, which is highly constrained in the medial ball-in-socket, it may be difficult to completely control tibiofemoral motion by geometry alone. Furthermore, our results do not dispel concerns about polyethylene wear and implant loosening during long-term follow-up. Postoperative satisfaction rated by KSS (2011) was significantly better in the MP-CS group than in the MP-PS group and was thought to reflect patients’ preference for the surface geometry of the MP-CS when performing activities of daily living that cause medial pivot.

## Data Availability

The datasets used and/or analyzed during the current study are available from the corresponding author on reasonable request.

## Abbreviations

3D: Three-dimensional
WBL ratio: Weight bearing line ratio
ACL: Anterior cruciate ligament
COR: Center of axial rotation
CS: Cruciate-substituting
FTA: Femorotibial angle
ICOR: Instantaneous center of axial rotation
KSS: Knee society score
MP: Medial pivot
PC: Point cluster
PCL: Posterior cruciate ligament
PS: Posterior-stabilized
pTEA: Projected transepicondylar axis
ROM: Range of motion
TEA: Transepicondylar axis
TKA: Total knee arthroplasty
